# Heat syndrome types prediction of traditional Chinese medicine in acute ischemic stroke through deep learning: a pilot study

**DOI:** 10.3389/fphar.2025.1601601

**Published:** 2025-08-04

**Authors:** Xiongwu Yu, Lingqian He, Qi Wang, Zhongyun Zhang, Huaiqiu Zhu, Juexian Song

**Affiliations:** ^1^ Department of Neurology, Xuanwu Hospital, Capital Medical University, Beijing, China; ^2^ Department of Biomedical Engineering, College of Future Technology, and Center for Quantitative Biology, Peking University, Beijing, China; ^3^ Academy for Advanced Interdisciplinary Studies, Peking University, Beijing, China; ^4^ Whiting School of Engineering, Johns Hopkins University, Baltimore, MD, United States

**Keywords:** deep learning, convolutional neural network, acute ischemic stroke, traditional Chinese medicine, integration of Chinese medicine and biomedicine

## Abstract

Integrating Chinese medicine and biomedicine for treating acute ischemic stroke (AIS) presents a promising strategy. Accurately predicting Traditional Chinese Medicine (TCM) heat syndrome types in AIS patients is crucial for guiding appropriate medication use within this combined treatment strategy. In this study, a clinical cohort including TCM syndromes, laboratory markers, and baseline assessments, were collected from 193 AIS patients. We developed a deep learning method with Convolutional Neural Networks (CNNs) to predict heat syndrome types in AIS patients by integrating TCM pattern characteristics and laboratory indicators. Feature importance was assessed using SHapley Additive exPlanations (SHAP) and permutation importance, and partial dependence plots (PDP) were used to explore the relationships between features and predictions. The model with the comprehensive feature dataset achieved an accuracy of 0.95, F1 score of 0.95, and AUC of 0.91 on the test set, exhibiting better performance overall compared to predictions based solely on TCM pattern characteristics or laboratory indicators. Key factors associated with the heat syndrome types included Tongue Teeth Marks, Stool, Sweat, Tongue Fissures, glycated hemoglobin (HbA1c), triglycerides (TG), fasting blood glucose (FBG) and total cholesterol (CHO). In conclusion, this study confirms the effectiveness of the CNN model in predicting heat syndrome types in AIS patients when incorporating TCM patterns with biochemical laboratory indicators.

## 1 Introduction

Acute Ischemic Stroke (AIS) constitutes the predominant type of stroke, accounting for about 70% of strokes (Wang et al., 2017). Although the standardized mortality rate for stroke exhibits a decline on a global scale from 1990 to 2015, there has been a concurrent escalation in the annual incidence of stroke cases, the number of associated deaths, and the loss of disability-adjusted life years (Tsao et al., 2022). In China, stroke has become the leading cause of death and disability, accounting for 22.3% of all deaths. Ischemic stroke dominates in all stroke cases, accounting for 69.6%–77.8% (Wang et al., 2017).

Stroke classification methodologies that are extensively employed in clinical practice include Trial of Org 10,172 in Acute Stroke Treatment (TOAST), Causative Classification System (CCS), and ASCOD (A for atherosclerosis, S for small vessel disease, C for cardiac source, O for other cause, D for dissection). Compared with TOAST, both CCS and ASCOD can reduce the number of patients classified as having undetermined etiology. The new classification can only fully consider each potential cause, reduce information loss, and more accurately determine stroke subtypes (Amarenco et al., 2013; Gökçal et al., 2017). However, the therapeutic approach to AIS predominantly relies on antiplatelet aggregation, thrombolysis, mechanical thrombectomy, and stroke care during hospitalization, lacking in individualized treatment. Traditional Chinese Medicine offers distinct advantages in personalized treatment strategies and taking into account a holistic view of patients’ factors. Therefore, the integration of TCM patterns with modern medical diagnosis and stratifying diseases offers innovative perspectives of diagnosis strategies and solutions for diseases management (Jiang et al., 2012).

Based on the previous Xuanwu classification (simplified classification), AIS patients can be stratified into four types: phlegm-heat pattern, phlegm-damp pattern, qi-deficiency pattern, and yin-deficiency pattern. In previous studies, we analyzed the laboratory indicators among different types of patients, and found that there were statistical differences among indicators such as low-density lipoprotein cholesterol (LDL-C), homocysteine (Hcy), and fibrinogen (Fib) (Song et al., 2019b). We also found that treatment based on classification can reduce the levels of plasma Fib, platelet aggregation test (PAgT), C-reactive protein (CRP), and trimethylamine oxide (TMAO) in AIS patients with heat pattern, as well as reduce the incidences of ischemic cerebrovascular events within 3 and 6 months after endovascular intervention examination (Song et al., 2019a). At the same time, in the study of gut microbiota, we found treatment after classification can decrease the lipopolysaccharide- (LPS-) producing bacteria, reduced genes on the biosynthesis of trimethylamine (TMA) and increase its decomposition genes. This leads to a reduction in the biosynthesis of LPS and trimethylamine N-oxide (TMAO), making a positive change in microbial diversity, and the gut microbial are closer to healthy individuals. Compared with only using the recommendations on guidelines, this integrated approach yields superior therapeutic outcomes (Lin et al., 2014; Guo et al., 2021).

Previous clinical studies have shown that TCM pattern differentiation can benefit AIS patients (Lin et al., 2014; Song et al., 2019a; Guo et al., 2021). However, the application of TCM into AIS treatment still faces several challenges. Firstly, the wide variety of TCM patterns and the intricacy of syndrome differentiation complicate the treatment process. Additionally, the complexity of the four TCM diagnostic methods and the reliance on practitioners’ individual experience, combined with the lack of objective standards recognized by both TCM and biomedicine, as well as the traditional view that separates TCM patterns from clinical biomedical indicators, all limit the integration of TCM into biomedical practice.

Recently, artificial intelligence methods have gradually been used to deal with clinical issues related to stroke (Heo et al., 2019; Fang et al., 2022; Lv et al., 2023) and assist traditional Chinese medicine diagnosis and treatment (Wang et al., 2020; Zhang et al., 2021; Liu et al., 2023; Lim et al., 2024; Pan et al., 2024; Zhou et al., 2024), but few studies have combined the diagnostic methods of TCM and biomedicine. To address these issues, this study builds on previous researches by simplifying the Xuanwu classification for AIS into two primary categories: heat patterns (including phlegm-heat and yin-deficiency types) and non-heat patterns (including phlegm-damp and qi-deficiency types), greatly reducing the complexity of syndrome differentiation (Cheng et al., 2013; Han et al., 2015; Song et al., 2019b; Zhang and Yang, 2023). Furthermore, we selected non-invasive, easily administered TCM observation and inquiry indicators for scoring and classification, combined with basic laboratory tests, to develop a Convolutional Neural Network model capable of accurately predicting the TCM heat syndrome types in AIS patients. This approach fosters a more seamless and effective convergence of TCM and clinical biomedical diagnostic methodologies, thereby enhancing the clinical applicability and precision of TCM pattern differentiation in the context of AIS management.

## 2 Materials and methods

### 2.1 Patients

Patients were diagnosed with AIS, and the TOAST (Trial of Org 10,172 in Acute Stroke Treatment) classification is the subtype of large-artery atherosclerosis in Xuanwu hospital, Capital Medical University.

Inclusion criteria: (1) Age ≥ 18 years. (2) Clinically diagnosed with acute ischemic stroke based on the following clinical criteria: Sudden onset of focal neurological deficits; Symptoms lasting for ≥ 24 h; Neuroimaging (MRI with DWI or CT) indicating focal cerebral ischemia or infarction; Exclusion of intracerebral hemorrhage or other non-vascular causes by MRI or CT. (3) The time from stroke onset to hospital admission is ≤ 14 days. If the exact onset time is unknown, the time of stroke onset is defined as the “last known well” time.

Exclusion criteria: Serious systemic diseases that may affect the outcome, such as liver and kidney-related diseases, hematological diseases, autoimmune diseases, malignant tumors, chronic severe infections, psychopathy, drug and alcohol abuse, and the like. Those with poor physical conditions and poor compliance will also be excluded. After applying the inclusion and exclusion criteria, 193 patients were included. The study was approved by the Ethics Committee of Xuanwu Hospital of Capital Medical University. The data are anonymous and from previous research, the requirement for informed consent was therefore waived.

### 2.2 Collection of laboratory indicators and pattern characteristics

Baseline information, including age, gender, medical history, NIHSS, Barthel index (BI), Glasgow coma scale (GCS), and modified Rankin scale (mRS), was collected by physicians on admission. All patients’ fasting venous blood were collected on the second morning of hospitalization, and laboratory indicators were tested (Supplementary Table S1). Other examinations include MRI, transcranial doppler (TCD), carotid ultrasonography, echocardiography, abdominal ultrasonography, and the medications used in the acute ischemic stroke patients, including the use of aspirin, clopidogrel, and statins.

The collection of TCM syndromes mainly relies on non-contact inspection and inquiry. Clinical physicians conducted a TCM symptom assessment by inquiring about patients’ bowel and urinary function, temperature sensitivity, sweating patterns, and dietary preferences. They observed and documented auscultation and olfactory information, including voice tone and breath odor. Following a standardized TCM data collection protocol, patients were asked to perform specific tasks, such as answering simple questions, blinking, smiling, extending the tongue, curling the tongue, and maintaining movement in the affected limbs for 5 s. Alongside collecting facial and tongue images, physicians also assessed the patient’s neurological function score.

The collected facial and tongue images were then scored and classified by a specialized integrative neurology team at Xuanwu Hospital. An initial classification was made by a resident physician, followed by independent reviews from two senior integrative medicine specialists who verify the scoring and provide final classifications. If discrepancies arise, the classification is further reviewed by a chief physician, and the expert panel discusses to reach a consensus on the final classification. The final classifications, including the simplified (Xuanwu) classification and heat syndrome types classification, were recorded (Supplementary Table S2).

### 2.3 Data pre-processing

For the pattern characteristics, the maximum proportion of missing data was 5.13%. Given this relatively small proportion, we chose to directly delete these entries. In the case of laboratory indicators, we only retained indicators with more than 170 non-missing values. The maximum proportion of missing data was 10.05%. Similarly, we opted to delete these entries. After missing value deletion and feature screening, we combined both pattern characteristics and laboratory indicators as comprehensive features to further observe, analyze, and compare the data.

Considering the complexity of clinical medical data distribution, we calculated the skewness of laboratory indicators data distribution. For indicators with skewness absolute value greater than 1.0, we applied Box-Cox transformation (Marimuthu et al., 2022).

We performed Chi-Square Tests between each feature and label (heat syndrome type). For laboratory indicators, we classified them into three categories: within the normal range, above the normal range, or below the normal range. For TCM pattern characteristics, we divided them into two categories according to whether the score was 0 or not, since a score of 0 indicated that the pattern was normal. The top 10 laboratory indicators and top 10 TCM pattern characteristics ranked by Chi-2 value were presented in Supplementary Table S4. Chi-Square Tests of sex, NIHSS score and mRS score were also performed.

### 2.4 Neural network model and feature importance analysis

The CNN was used to predict heat syndrome types in AIS patients as a binary classification task, differentiating between heat pattern and non-heat pattern. Due to the imbalanced nature of the original dataset (comprehensive features of heat pattern 75%, non-heat pattern 25%), we employed random oversampling to achieve a balanced 1:1 ratio, then randomly divide it into training and test sets at the ratio of 8:2. The model was trained using a Leave-one-out cross-validation (LOOCV) approach, iteratively validating with one sample while the rest formed the training set. The model was then re-trained on the full training dataset and evaluated on an independent test set, demonstrating its effectiveness in accurately predicting heat syndrome types and supporting integrated Chinese medicine and biomedicine diagnosis in AIS patients.

We trained and tested CNN models using laboratory indicators data, TCM pattern characteristics data, and comprehensive features data, respectively. The comprehensive features data integrated the first two types of data. The CNN architecture comprised two convolutional layers: the first with a kernel size of 3, followed by ReLU activation and max-pooling, and the second a kernel size of 2, also followed by ReLU activation and max-pooling. The output was then flattened and passed through a fully connected layer, with a sigmoid activation function providing classification probabilities. The receiver operating characteristic (ROC) curves curve was presented to visualize the model’s discriminative ability between the heat and non-heat patterns in AIS patients. The SHAP was used to evaluate the importance of features in predicting results and analyze the importance of each concrete feature (Lundberg et al., 2020). We analyzed and screened out the 20 most important features that significantly impact on predicting heat syndrome classification results. Furthermore, permutation feature importance was utilized to assess the importance of each feature in the CNN model. This method involves shuffling the values of individual features and observing the resulting change in model accuracy. A significant drop in accuracy suggests that the shuffled feature is highly important, as the model’s ability to make accurate predictions is disrupted. To observe the relationships between TCM patterns and laboratory indicators and their predicted targets, partial dependence plots (PDP) were constructed for the several most important features (Zhao and Hastie, 2021).

## 3 Results

### 3.1 Baseline patient characteristics

#### 3.1.1 Preprocessed dataset characteristics

After missing value deletion and feature screening, we ultimately retaining 123 data points for analysis, including 22 TCM pattern characteristics and 31 laboratory indicators. Before applying the Box-Cox transformation, approximately 64.5% (20 out of 31) of the laboratory indicators exhibited significant skewness (skewness > 1.0 or <−1.0), which could negatively affect the prediction of heat syndrome types. After the transformation, only the skewness of anti-thyroglobulin antibodies (TG-Ab) was slightly greater than 1.0 (skewness = 1.18) (Supplementary Table S3). Several laboratory indicators and TCM pattern characteristics demonstrated significant associations (p-value <0.05) with heat syndrome, whereas sex, NIHSS score, and mRS score showed no significant association (Supplementary Table S4).

#### 3.1.2 Distribution characteristics of patients’ gender

**FIGURE 1:**
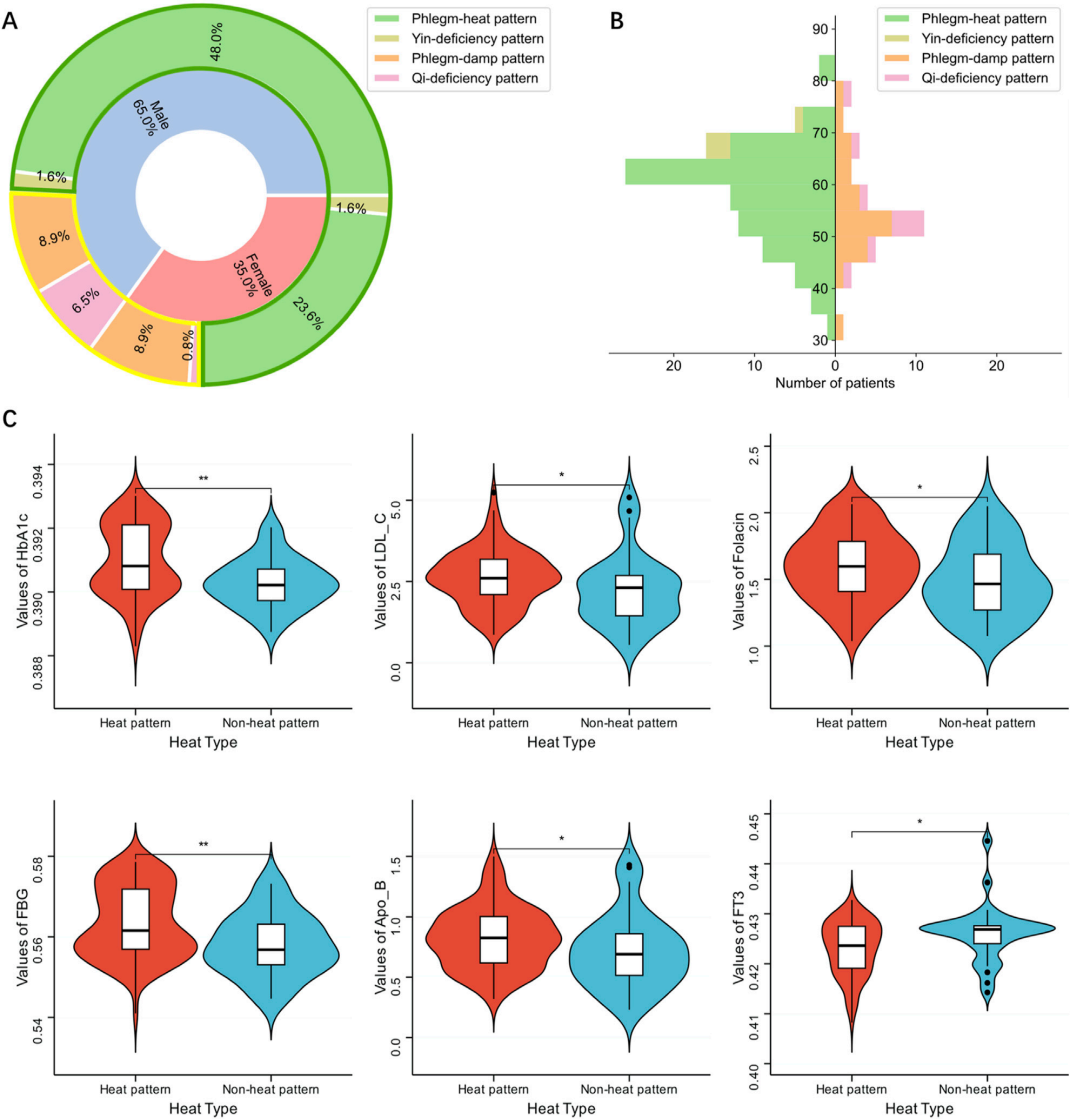
Characteristics of patient data. **(A)** Gender composition of simplified classifications and heat syndrome types of patients. The circular area surrounded by green lines indicates heat pattern, and the area surrounded by yellow lines indicates non-heat pattern. **(B)** Age structure of simplified classifications and heat syndrome types of patients. The left side of the age axis represents the heat pattern, and the right side represents the non-heat pattern. **(C)** Distribution of laboratory indicators in patients with heat pattern and non-heat pattern. Only indicators with statistically significant differences between the two are shown.

In the simplified (Xuanwu) classification, more male patients were included in the experiment than female patients. The proportion of males classified under the phlegm-heat pattern was markedly higher than that of females. Conversely, females exhibited a higher prevalence in the phlegm-damp and qi-deficiency patterns compared to males (Li et al., 2008), with the phlegm-damp pattern being particularly more common among females. Within the male cohort, the phlegm-heat pattern predominated significantly over the other patterns, whereas the yin-deficiency pattern remained the least prevalent among both genders (Figure 1A). Notably, in males, the phlegm-damp pattern was significantly less common than the qi-deficiency pattern, while in females, the opposite trend was observed, with the phlegm-damp pattern surpassing the qi-deficiency pattern.

No significant gender distribution disparity was observed between the heat and non-heat patterns in the classification based on heat syndrome type (Chi-Square Test p-value 0.773) (Supplementary Table S4). The proportion of males classified under the heat pattern was slightly higher than that of females, while the non-heat pattern was marginally more common among females.

#### 3.1.3 Distribution characteristics of patients’ age

The age distribution of the phlegm-heat pattern and phlegm-damp pattern in the simplified (Xuanwu) classification spanned a wide range, from 30 to 85 years old. The peak of the phlegm-heat pattern was concentrated between 60 and 65 years old, while the phlegm-damp pattern was concentrated between 50 and 55 years old. Yin-deficiency and qi-deficiency patterns had a relatively smaller distribution range. The qi-deficiency pattern tends to be found in younger individuals, mainly distributed between 40 and 60 years old, while the yin-deficiency pattern tended to be found in older individuals, distributed between the ages of 65 and 75 (Figure 1B).

In heat syndrome types classification, both heat pattern and non-heat pattern exhibited a broad age distribution with a peak between 50 and 65 years old. However, patients with heat pattern were predominantly between 60 and 65 years old, while non-heat pattern patients were concentrated between 50 and 55. Consequently, the aging of AIS patients with heat pattern was more evident than non-heat pattern (Figure 1B).

#### 3.1.4 Distribution characteristics of patients’ laboratory indicators

From heat syndrome types classification, the distribution of HbA1c, LDL-C, Folacin, FBG, apolipoprotein B (Apo-B) and free triiodothyronine (FT3) were significantly different (Figure 1C; Supplementary Table S4). These indicators suggested that AIS patients have significant differences in heat syndrome types classification. The distribution of the remaining 25 indicators was similar and has no significant difference (Supplementary Figure S1).

#### 3.1.5 Correlations between laboratory indicators and TCM pattern characteristics data

**FIGURE 2:**
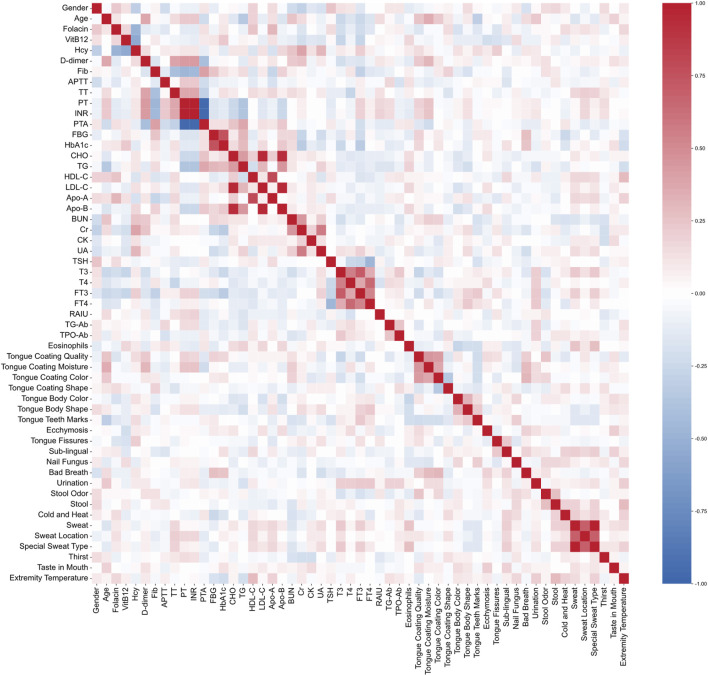
Pearson correlation plot of all the features. Red indicates a positive correlation, and blue indicates a negative correlation. The stronger the correlation, the darker the color.

The correlations among the selected features held great importance in understanding the intrinsic relationships and interactions within the prediction model. We assessed the correlations between TCM pattern characteristics data and laboratory indicators data (Figure 2). The correlation coefficients for prothrombin activity (PTA), international normalized ratio (INR) and prothrombin time (PT) exceeded 0.96, with INR showing a positive correlation with PT and PTA displaying negative correlations with both INR and PT. Similarly, the correlation coefficients between CHO, LDL-C and Apo-B exceeded 0.94, and those between high-density lipoprotein cholesterol (HDL-C) and Apo-A exceeded 0.79. In addition, the correlation between HbA1c and FBG reached 0.75. Strong correlations were also observed among triiodothyronine (T3), thyroxine (T4), FT3, and free thyroxine (FT4), as well as among Sweat, Sweat Location, and Special Sweat Type. Apart from these features, no significant correlations were found among the remaining variables.

### 3.2 Prediction model of TCM heat syndrome types classification of AIS by deep learning

#### 3.2.1 Model prediction effect of laboratory indicators data

**FIGURE 3:**
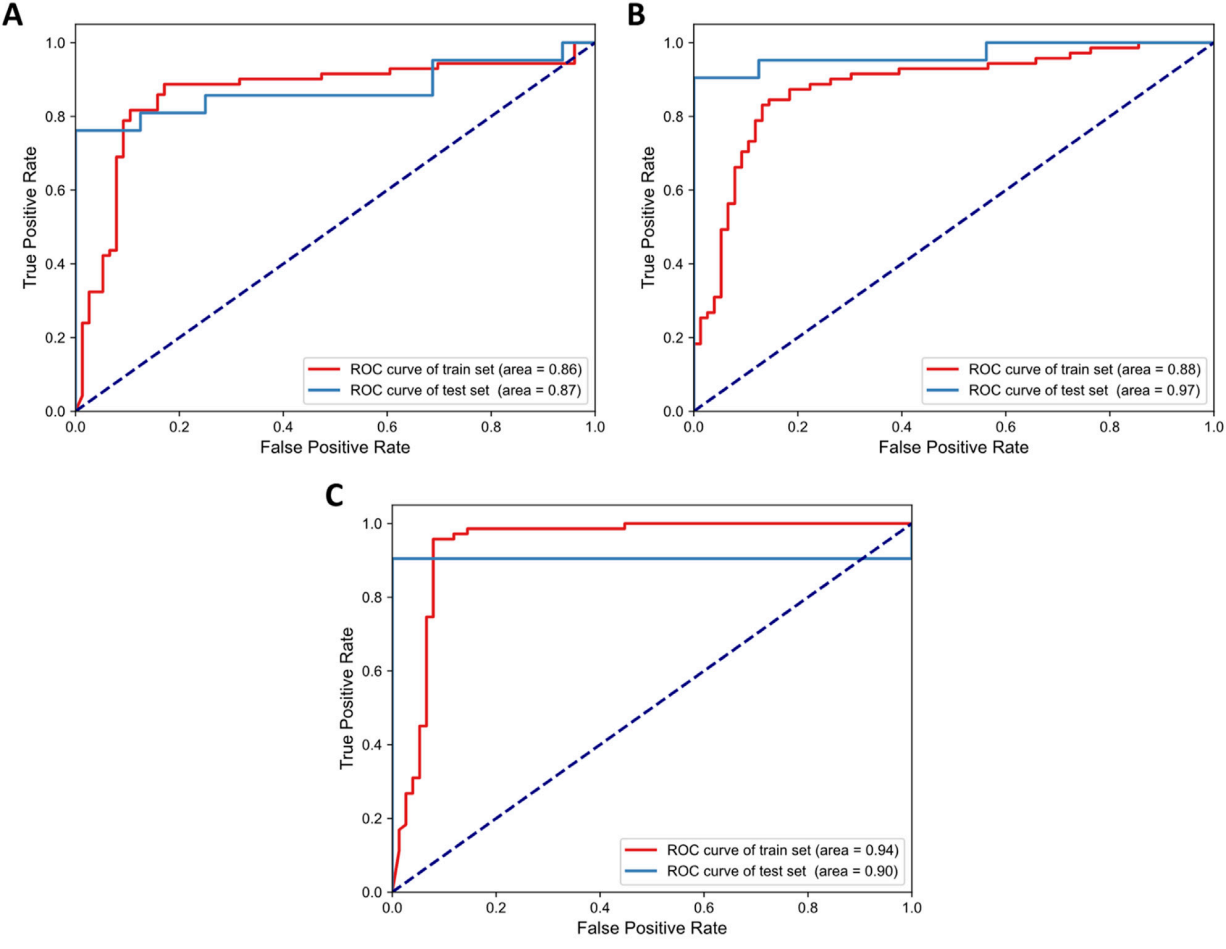
ROC curves of the CNN model’s performance across three different datasets: **(A)** laboratory indicators, **(B)** TCM pattern characteristics, and **(C)** comprehensive features.

In the evaluation of the model prediction performance using laboratory indicators data, the CNN model was tested using LOOCV during the training phase. The CNN model demonstrated a moderate ability to predict outcomes, achieving an accuracy of 0.80, an F1 score of 0.77, and an AUC of 0.86. When applied to an independent test dataset, the CNN model exhibited improved performance with an accuracy of 0.78, an F1 score of 0.77, and an AUC of 0.87 (Figure 3A). Sensitivity, specificity, positive predictive value (PPV), and negative predictive value (NPV) were presented in Supplementary Table S5.

#### 3.2.2 Model prediction effect of TCM pattern characteristics data

In our evaluation of the model prediction performance using TCM pattern characteristics data, the CNN model was assessed during the training phase using LOOCV and on an independent test dataset. During the training phase, the CNN model demonstrated a robust ability to predict TCM pattern characteristics, achieving an accuracy of 0.81, an F1 score of 0.79, and an AUC of 0.88. When applied to the independent test dataset, the CNN model maintained its performance with an accuracy of 0.84, an F1 score of 0.83, and an AUC of 0.97 (Figure 3B). Sensitivity, specificity, PPV, and NPV were presented in Supplementary Table S5. These results highlighted the model’s consistent effectiveness in predicting TCM syndromes, particularly reflected in its high AUC value.

#### 3.2.3 Model prediction effect of comprehensive features data

We compared the comprehensive feature performance of the CNN model. For the training phase, the CNN model with LOOCV achieved an accuracy of 0.88, an F1 score of 0.88, and an AUC of 0.94. When evaluated on the test dataset, the model demonstrated strong performance with an accuracy of 0.95, an F1 score of 0.95, and an AUC of 0.91 (Figure 3C). This was the only method with all three metrics above 0.9 on the test set. Sensitivity, specificity, PPV, and NPV were presented in Supplementary Table S5. With the exception of AUC on the test set, the model utilizing comprehensive features achieved superior or comparable performance across all other evaluated metrics.

The above results highlighted the CNN model’s exceptional predictive capability when integrating comprehensive feature data, particularly evident in the high accuracy and F1 score achieved on the test dataset. TCM pattern characteristics exhibited overall higher accuracy, F1 score, and AUC compared to predictions based solely on laboratory indicators, indicating that these characteristics were particularly effective in classifying heat syndrome types in AIS patients. Notably, TCM pattern characteristics achieved the highest AUC score of 0.97, suggesting they captured subtle variations that enhance sensitivity. These findings suggested that while the comprehensive dataset, including both TCM pattern characteristics and laboratory indicators, offered the most robust approach for the auxiliary diagnosis of AIS, TCM pattern characteristics alone were also capable of predicting heat syndrome types.

### 3.3 The main factors affecting the classification of TCM heat types in AIS

In the application of CNN model for predicting TCM heat types in AIS patients, the significance of features was determined using SHAP values, which provided a clear interpretation of the model’s predictions by highlighting the contribution of each feature. Those features with the highest SHAP values indicated their significant influence on the model’s ability to classify patients into heat or non-heat patterns.

**FIGURE 4:**
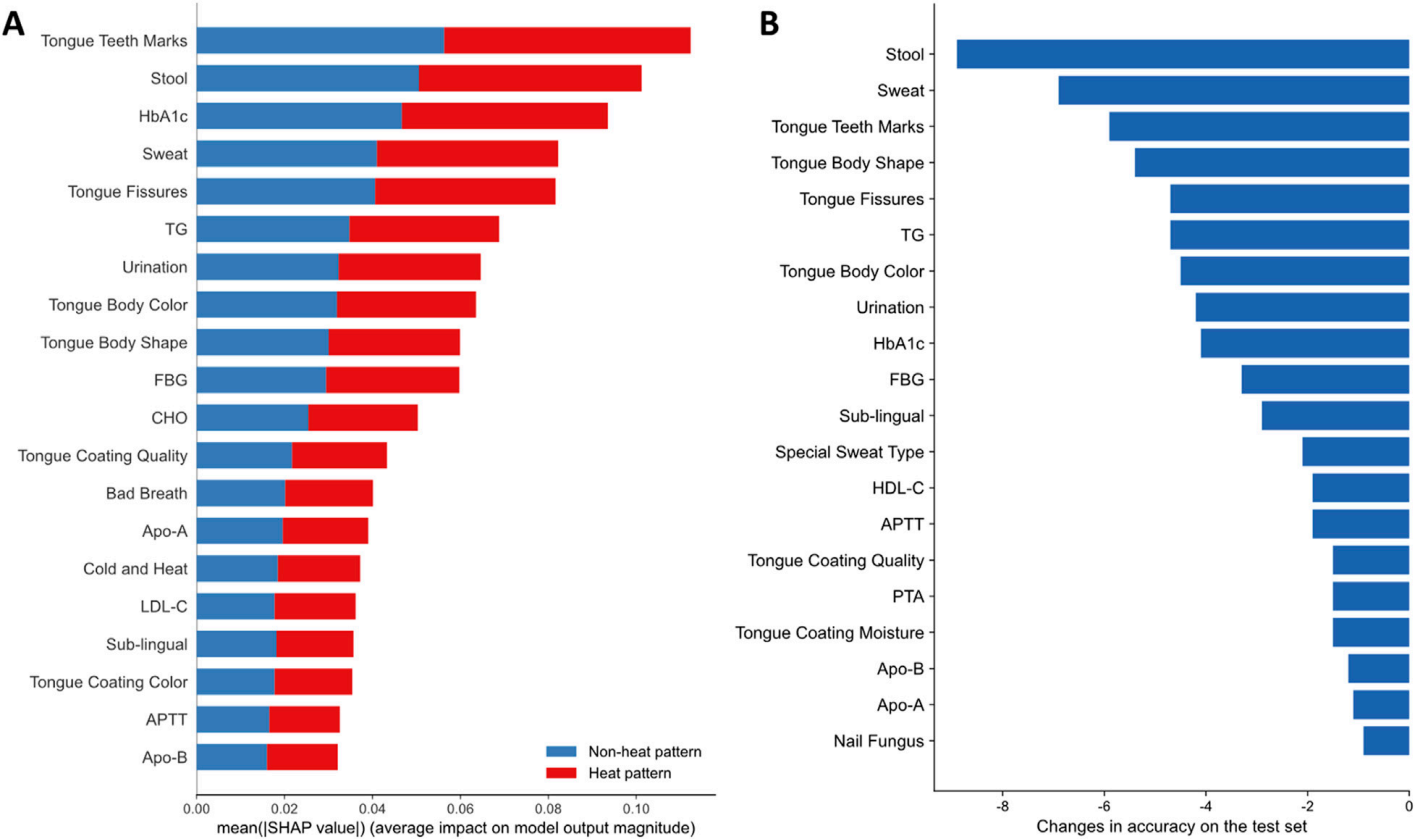
The importance of features with comprehensive dataset on the prediction results. **(A)** Feature importance of the top 20 features using the average of the absolute SHAP values of two classes and ranking features in order of importance. Blue represents non-heat pattern, and red represents heat pattern; **(B)** The changes in accuracy on the test set based on permutation feature importance. The 20 features that caused the most decrease in accuracy are shown.

Among the laboratory indicators, the top five influential features were CHO, HbA1c, LDL-C, Hcy and uric acid (UA) (Supplementary Figure S2A). For the TCM pattern characteristics, the five most critical features were Stool, Cold and Heat, Tongue Body Shape, Sweat, Tongue Teeth Marks (Supplementary Figure S2B). When using the comprehensive dataset, which integrates both TCM pattern characteristics and laboratory indicators, the top critical indicators were Tongue Teeth Marks, Stool, HbA1c, Sweat, Tongue Fissures and TG, among which four indicators demonstrated their consistent relevance across different analyses (Figure 4A).

Based on the permutation feature importance algorithm, we identified the top 20 features that caused the most decrease in accuracy on the test set (Figure 4B). A decrease in accuracy upon feature permutation suggested that the feature is crucial for improving model performance. After shuffling the data distribution of Stool, the accuracy of the model dropped the most, indicating that this feature had the most significant impact on model performance. It is worth mentioning that several indicators such as Stool, Sweat, Tongue Teeth Marks, Tongue Fissures, TG and HbA1c, were identified as important features affecting the model performance by both SHAP-based and permutation-based methods. This consensus underscored their critical role in the classification of heat syndrome types.

### 3.4 Correlations between main factors

**FIGURE 5:**
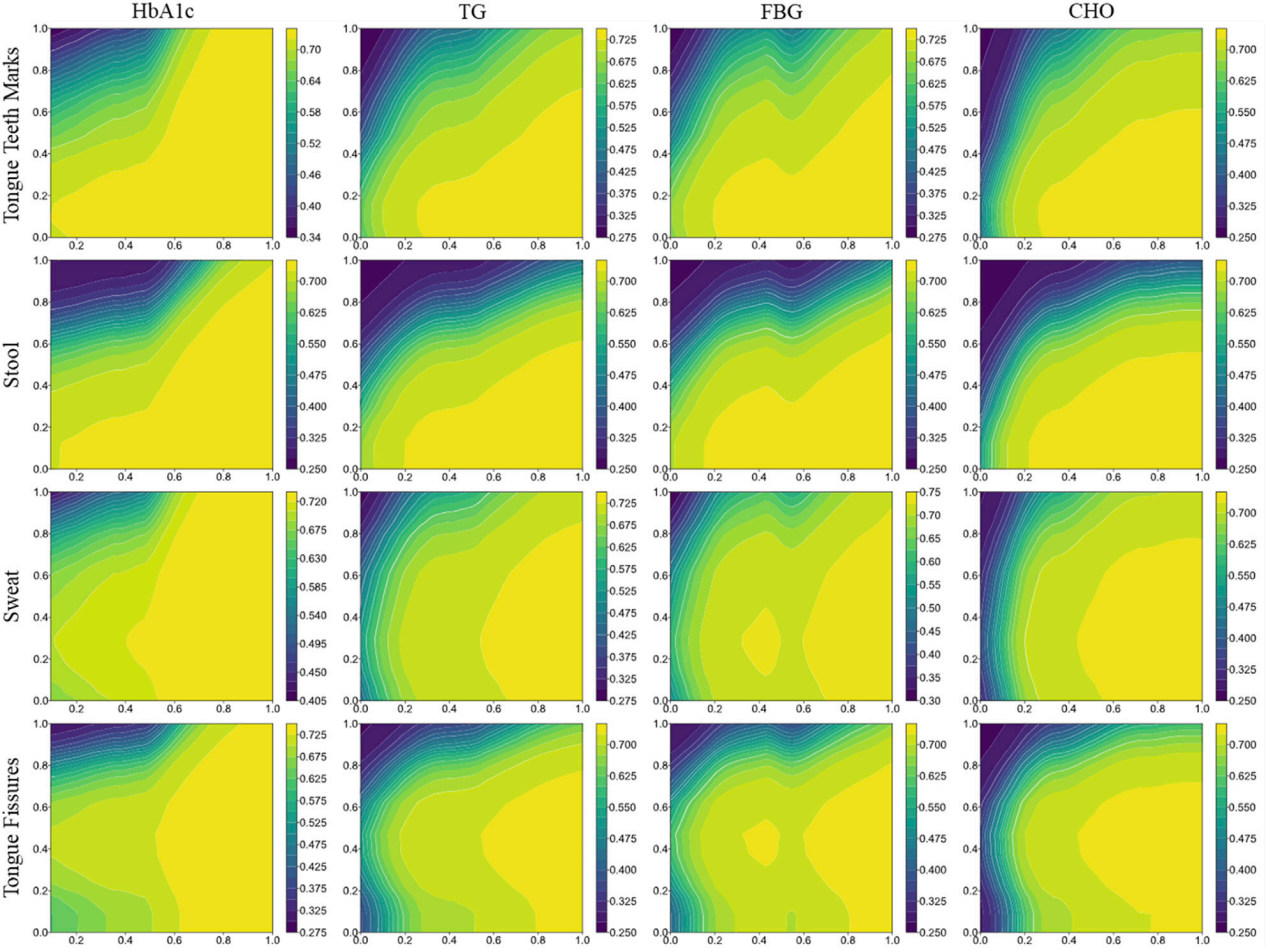
Two-way partial dependence plots of the most important features in TCM patterns and laboratory indicators. Two-way plots combine two features from TCM patterns and laboratory indicators respectively (y and x-axis), showing how a simultaneous change of values influence the prediction performance.

We observed that the order of feature importance shifted when employing the comprehensive dataset to predict heat syndrome types, as opposed to predictions using only TCM pattern or laboratory indicators. This suggested that the integration of these two parts of dataset results in interactive effects, thereby altering their relative importance in the model’s prediction accuracy. Consequently, we drew the two-way partial dependence plots of the most important features in TCM patterns and laboratory indicators (Figure 5). It was obvious that there were additive or synergistic interactions between TCM patterns and laboratory indicators, especially for Tongue Teeth Marks and Stool with HbA1c, TG, FBG, which were the most ranked TCM patterns and laboratory indicators respectively. The synergy effect between them may be one of the reasons for the improved model performance with comprehensive dataset.

## 4 Discussion

In this study, leveraging extensive clinical experience, we formulated a strategy to simplify AIS into heat and non-heat patterns based on Xuanwu classification, predicated on the foundation of accurate diagnosis. By utilizing deep learning model to identify key indicators affecting classification, this study explored clinically meaningful laboratory markers and developed a diagnosis and treatment plan that can be consistently applied by both Chinese medicine and biomedicine practitioners. Through this approach, clinicians can classify acute stroke patients, guide clinical decisions, and improve patient symptoms based on simple consultation and inspection combined with essential laboratory indicators. This diagnostic model can significantly enhance diagnostic efficiency in outpatient settings through rapid assessment. It also provides a valuable tool for expediting diagnosis and guiding treatment for acute stroke patients in emergency care, thereby improving outcomes, and helps standardize classification and clinical decision-making for hospitalized patients.

During the modeling phase, we developed a CNN model to predict patients’ heat syndrome types based on the (non-) heat pattern classification corresponding to the four categories of the simplified (Xuanwu) classification. CNN is able to automatically capture nonlinear interactions and local patterns within structured data through convolutional layers. Previous studies have employed CNN to predict clinical outcomes such as in-hospital mortality using structured data (Zhang et al., 2020). Given that heat syndrome types may correspond to specific combinations of TCM patterns (e.g., tongue characteristics) and laboratory indicators, we employed CNN to integrate Chinese medicine and biomedicine indicators. The binary classification of heat and non-heat syndrome types over the original four TCM patterns was consistent with the simplified classification system commonly used at Xuanwu Hospital (Song et al., 2019b). This approach not only improved the balance of the dataset, thereby enhancing the robustness and predictive accuracy of the model, but also offered greater clinical applicability. The results of model prediction suggest that the integration of TCM pattern characteristics and laboratory indicators is more effective for AIS classification. HbA1c, TG, CHO, and FBG are several essential indicators for classifying heat syndrome types. Elevated HbA1c and TG levels are both linked to an increased risk of stroke, as well as a higher likelihood of stroke recurrence and mortality in stroke patients (Rozanski et al., 2014; Mitsios et al., 2018; Bhatt et al., 2019; Zhou et al., 2020; Bao and Gu, 2021; Huang et al., 2022; Yang et al., 2023). CHO is considered to be a significant independent predictor of stroke severity and poor outcomes in patients with AIS (Zhao et al., 2016; Chen et al., 2017; Niu et al., 2020). Some studies have even shown an inverse and almost linear association between CHO and mortality after stroke (Olsen et al., 2007). FBG is closely related to ischemic stroke, especially in diabetic patients (Tanne et al., 2004; Boden-Albala et al., 2008), and is also considered a predictor of progressive infarction in men with AIS (Yu et al., 2022). From a TCM perspective, internal heat can gradually deplete body fluids, impairing the spleen and stomach’s digestive and absorptive functions, which may lead to metabolic disruptions, particularly in blood glucose and lipid levels. Furthermore, heat damages yin, leading to a yin-yang imbalance that further impacts metabolic processes. Previous studies have shown that patients with heat syndrome may have higher blood glucose and lipid levels (Mierzecki et al., 2013; Gu et al., 2019). In addition, LDL-C and Hcy are also critical indicators according to predictions based solely on laboratory indicators. High LDL-C levels are positively correlated with the risk of atherosclerosis, a major contributor to ischemic stroke (Gu et al., 2019; Bohula et al., 2024). Hcy, on the other hand, increases oxidative stress, triggers vascular inflammation, damages endothelial cells, and stimulates vascular smooth muscle cell proliferation, significantly elevating stroke risk (Dardik et al., 2000; Mierzecki et al., 2013; Kwon et al., 2014). Consequently, these laboratory indicators are significant in diagnosing and assessing heat syndrome in TCM.

Moreover, the model developed in this study serves as a conduit to unravel the intricate “disease-syndrome-symptom” relationships in TCM theory, offering a novel perspective on the diagnostic and therapeutic principles of TCM. This study not only provides a systematic approach of Xuanwu classification in AIS but also contributes to the scientific validation of TCM’s holistic views on health and disease, thereby bridging the gap between empirical knowledge and evidence-based practice in integrated Chinese medicine and biomedicine. For example, the CNN model validated a critical TCM theory by demonstrating that both Tongue Teeth Marks and Tongue Fissures are significant indicators for distinguishing heat from non-heat patterns in AIS patients. According to TCM theory, Tongue Teeth Marks is often related to spleen deficiency, yang vacuity with cold dampness, phlegm and retained fluid, and blood stasis (Wang et al., 2020; Tian et al., 2024). The diagnosis of tooth-marked tongue can greatly contribute to the symptom differentiation and treatment selection (Li and Dong, 2016). When spleen qi is insufficient, these functions are impaired, leading to dampness retention and tongue swelling, which forms teeth marks (Li et al., 2008). Prolonged dampness may further transform into damp-heat. Similarly, Tongue Fissures are explained in TCM as a result of “heat damaging yin”, where excessive heat depletes body fluids, leaving the tongue insufficiently nourished and causing fissures. Clinical studies have shown that tongue fissures are closely associated with inflammatory markers, suggesting their potential to reflect pathological processes such as inflammation or metabolic disorders (Kullaa-Mikkonen, 1988; Jiang et al., 2022). It is worth mentioning that Tongue Teeth Marks and Tongue Fissures are also the most concerned features of tongue diagnosis based on image recognition (Li et al., 2018; Weng et al., 2021; Yan et al., 2023; Zhang et al., 2025) and were both found to be closely related to disorders related to metabolic or cardiovascular health such as hypertension, dyslipidemia, and nonalcoholic fatty liver disease (Jiang et al., 2022). The CNN model also highlighted stool characteristics as key indicators of internal heat. In TCM, patients with heat patterns often present with constipation or dry stools (Shen et al., 2017), as internal heat disrupts water metabolism, causing intestinal dryness. Therefore, stool characteristics, particularly constipation, not only aid in distinguishing heat from non-heat patterns but also provide valuable insights into the body’s metabolic state, making them a crucial variable in predictive models.

One primary limitation of this study is the small sample size which may affect the model’s generalizability and robustness. Therefore, we can regard this study as a pilot study, exploring the feasibility of using deep learning models, particularly CNN to integrate TCM and laboratory features for heat pattern prediction. Additionally, the imbalanced distribution of TCM pattern types necessitated resampling, potentially introducing bias and reducing predictive validity. Although directly deleting missing values ​​has achieved good results in our study, other data processing methods are also worth exploring when facing larger amounts of data. More rigorous and standardized methods are also needed to reduce missing information in data collection. Future studies should include larger, multi-center patient cohorts to expand sample size and enhance generalizability, which will allow for more robust statistical analysis and the development of more generalizable and powerful predictive models, including advanced deep learning approaches. Furthermore, for clinical application, it is essential to train healthcare professionals in using and interpreting the CNN model that integrates TCM and laboratory data. The insights from this pilot will also guide us in potentially refining and prioritizing the most impactful TCM pattern characteristics and laboratory indicators for future, larger-scale investigations.

## 5 Conclusion

In conclusion, this study demonstrates that deep learning, specifically through CNN model, is capable of effectively predicting heat syndrome types in AIS patients by integrating TCM pattern characteristics and laboratory indicators. Moving forward, the next step should involve conducting large-scale, multi-center clinical trials to validate these findings across diverse patient populations. Such trials would not only enhance the model’s generalizability but also refine its diagnostic accuracy and clinical utility. Additionally, future research should explore the integration of this predictive model into electronic health records systems, facilitating real-time, standardized diagnostics and potentially improving clinical decision-making processes. This progression holds significant potential for advancing the integration of Chinese medicine and biomedicine, ultimately leading to more personalized and effective patient care.

## Data Availability

The raw data supporting the conclusions of this article will be made available on request from the corresponding author. The data are not publicly available due to privacy or ethical restrictions.
