# Supplementary material for: Heat syndrome types prediction of traditional Chinese medicine in acute ischemic stroke through deep learning: a pilot study
Source: Front Pharmacol. 2025 Aug 4;16:1601601. doi: 10.3389/fphar.2025.1601601 (PMC12358418; doi:10.3389/fphar.2025.1601601)
Supplement: Supplementary file 1 [file Supplementaryfile1.docx]

Supplementary Material

# Supplementary Tables and Figures

## Supplementary Tables

**Supplementary Table 1.** Laboratory indicators collected in this study.

| **Testing Program** | **Laboratory indicators** |
| --- | --- |
| Complete blood count (CBC) | Eosinophils; Basophils; Eosinophil ratio; Basophil ratio; Eosinophil percentage; Basophil percentage |
| Blood biochemical | Total cholesterol (CHO); Triglycerides (TG); High-density lipoprotein cholesterol (HDL-C); Low-density lipoprotein cholesterol (LDL-C); Apolipoprotein A (Apo-A); Apolipoprotein B (Apo-B); Creatinine (Cr), Uric acid (UA); Blood urea nitrogen (BUN); Fasting blood glucose (FBG); Glycated hemoglobin (HbA1c) |
| Coagulation tests include | D-dimer; Fibrinogen (Fib); Activated partial thromboplastin time (APTT); Thrombin time (TT); Prothrombin time (PT); International normalized ratio (INR); Prothrombin activity (PTA) |
| Thyroid function | Thyrotropin (TSH); Triiodothyronine (T3); Thyroxine (T4); Free triiodothyronine (FT3); Free thyroxine (FT4); Radioactive iodine uptake rate (RAIU); Anti-thyroglobulin antibodies (TG-Ab); Thyroid peroxidase antibody (TPO-Ab) |
| Immune-related test | Immunoglobulin G (IgG); IgA; IgM; Complement 3 (C3); C4 |
| Other tests | C-reactive protein (CRP); Hypersensitive CRP (hs-CRP); Creatine kinase (CK); Homocysteine (Hcy); Folacin; Vitamin B12 (VitB12); Urine pH |

**Supplementary Table 2.** TCM pattern characteristics evaluated in this study.

| **Simplified classification** | **Pattern characteristics** | **Heat type** |
| --- | --- | --- |
| Phlegm‐heat pattern | Yellow thick coating, lack of saliva, dryness of the mouth, bitter taste in the mouth, sticky and greasy sensation in the mouth, thirst and desire cold drinks, halitosis, fear of heat, dry stool and stinky; | Heat pattern |
| Yin-deficiency pattern | Thin tongue, crimson tongue, thin or peeled coating, tidal fever, night sweats, thirst, and desire cold drinks; |  |
| Phlegm-damp pattern | White and moist coating, enlarged tongue, teeth-marked tongue, bland taste in the mouth, thirst without a desire to drink, cold of the hands and feet, ungratifying defecation, sticky stool; | Non-heat pattern |
| Qi-deficiency pattern | Dusky tongue, white coating, tongue with/without teeth-marked, profuse sweating, fear of cold, bland taste in the mouth, dryness of the mouth, cold of the hands and feet, thin and unformed stool, incomplete defecation; |  |

**Supplementary Table 3.** Skewness of laboratory indicators data before and after Box-Cox transformation.

| Laboratory indicators | Skewness | |  | Laboratory indicators | Skewness | |
| --- | --- | --- | --- | --- | --- | --- |
|  | Before Box-Cox | After Box-Cox |  |  | Before Box-Cox | After Box-Cox |
| Folacin | 1.23 | 0.04 |  | Apo-A | 0.55 | 0.55 |
| VitB12 | 1.78 | -0.01 |  | Apo-B | 0.37 | 0.37 |
| Hcy | 2.54 | 0.03 |  | BUN | 1.38 | 0.03 |
| D-dimer | 5.06 | 0.27 |  | Cr | 2.34 | -0.02 |
| Fib | 0.30 | 0.30 |  | CK | 5.80 | -0.04 |
| APTT | 0.69 | 0.69 |  | UA | 0.40 | 0.40 |
| TT | 4.28 | -0.07 |  | TSH | 3.19 | -0.04 |
| PT | 0.56 | 0.56 |  | T3 | 4.68 | -0.02 |
| INR | 0.57 | 0.57 |  | T4 | 1.71 | -0.04 |
| PTA | 0.99 | 0.99 |  | FT3 | 7.47 | -0.16 |
| FBG | 1.46 | 0.20 |  | FT4 | 3.22 | -0.12 |
| HbA1c | 1.45 | 0.19 |  | RAIU | -0.10 | -0.10 |
| CHO | 0.48 | 0.48 |  | TG-Ab | 5.18 | 1.18 |
| TG | 1.70 | 0.06 |  | TPO-Ab | 7.11 | 0.53 |
| HDL-C | 1.52 | 0.06 |  | Eosinophils | 1.65 | 0.15 |
| LDL-C | 0.40 | 0.40 |  |  |  |  |

**Supplementary Table 4.**​​ Sex, NIHSS score, mRS score, top 10 laboratory indicators and top 10 TCM pattern characteristics ranked by Chi-2 value, were stratified by heat type after stroke.

| **Characteristic** | **Heat pattern (92)** | **Non-heat pattern (31)** | **P value** | **Chi-2 value** |
| --- | --- | --- | --- | --- |
| **Sex** |  |  | **0.773** | **0.083** |
| Male | 61 | 19 |  |  |
| Female | 31 | 12 |  |  |
| **NIHSS score** |  |  | **0.972** | **0.001** |
| <5 | 29 | 9 |  |  |
| 5~15 | 63 | 22 |  |  |
| **mRS score** |  |  | **0.962** | **0.077** |
| 0~1 | 35 | 11 |  |  |
| 2~3 | 35 | 12 |  |  |
| 4~5 | 22 | 8 |  |  |
| **FT3** |  |  | **0.036** | **6.631** |
| ↑ | 0 | 2 |  |  |
| -- | 85 | 28 |  |  |
| ↓ | 7 | 1 |  |  |
| **LDL-C** |  |  | **0.070** | **5.310** |
| ↑ | 25 | 4 |  |  |
| -- | 44 | 13 |  |  |
| ↓ | 23 | 14 |  |  |
| **CHO** |  |  | **0.077** | **5.130** |
| ↑ | 4 | 3 |  |  |
| -- | 70 | 17 |  |  |
| ↓ | 18 | 11 |  |  |
| **HbA1c** |  |  | **0.025** | **5.015** |
| ↑ | 47 | 8 |  |  |
| -- | 45 | 23 |  |  |
| **Apo-B** |  |  | **0.146** | **3.852** |
| ↑ | 9 | 3 |  |  |
| -- | 61 | 15 |  |  |
| ↓ | 22 | 13 |  |  |
| **Fib** |  |  | **0.155** | **3.732** |
| ↑ | 23 | 4 |  |  |
| -- | 65 | 27 |  |  |
| ↓ | 4 | 0 |  |  |
| **Folacin** |  |  | **0.167** | **3.583** |
| ↑ | 8 | 1 |  |  |
| -- | 80 | 26 |  |  |
| ↓ | 4 | 4 |  |  |
| **FBG** |  |  | **0.253** | **2.749** |
| ↑ | 41 | 9 |  |  |
| -- | 50 | 21 |  |  |
| ↓ | 1 | 1 |  |  |
| **UA** |  |  | **0.340** | **2.157** |
| ↑ | 12 | 2 |  |  |
| -- | 77 | 29 |  |  |
| ↓ | 3 | 0 |  |  |
| **TT** |  |  | **0.346** | **2.125** |
| ↑ | 5 | 0 |  |  |
| -- | 86 | 31 |  |  |
| ↓ | 1 | 0 |  |  |
| **Tongue Coating Color** |  |  | **0.007** | **7.330** |
| 0 | 9 | 10 |  |  |
| >0 | 83 | 21 |  |  |
| **Tongue Coating Quality** |  |  | **0.007** | **7.267** |
| 0 | 15 | 13 |  |  |
| >0 | 77 | 18 |  |  |
| **Extremity Temperature** |  |  | **0.029** | **4.741** |
| 0 | 86 | 24 |  |  |
| >0 | 6 | 7 |  |  |
| **Tongue Teeth Marks** |  |  | **0.036** | **4.407** |
| 0 | 73 | 18 |  |  |
| >0 | 19 | 13 |  |  |
| **Nail Fungus** |  |  | **0.036** | **4.375** |
| 0 | 89 | 26 |  |  |
| >0 | 3 | 5 |  |  |
| **Bad Breath** |  |  | **0.063** | **3.458** |
| 0 | 70 | 29 |  |  |
| >0 | 22 | 2 |  |  |
| **Tongue Coating Moisture** |  |  | **0.106** | **2.617** |
| 0 | 48 | 22 |  |  |
| >0 | 44 | 9 |  |  |
| **Taste in Mouth** |  |  | **0.316** | **1.005** |
| 0 | 54 | 22 |  |  |
| >0 | 38 | 9 |  |  |
| **Tongue Body Color** |  |  | **0.341** | **0.907** |
| 0 | 3 | 3 |  |  |
| >0 | 89 | 28 |  |  |
| **Urination** |  |  | **0.457** | **0.554** |
| 0 | 86 | 27 |  |  |
| >0 | 6 | 4 |  |  |

**Supplementary Table 5.** Performance comparison of models with different features.

|  |  | Accuracy | F1 score | AUC | Sensitivity | Specificity | PPV | NPV |
| --- | --- | --- | --- | --- | --- | --- | --- | --- |
| LOOCV | Laboratory indicators | 0.80 | 0.77 | 0.86 | 0.69 | 0.91 | 0.88 | 0.76 |
|  | TCM pattern characteristics | 0.81 | 0.79 | 0.88 | 0.73 | 0.88 | 0.85 | 0.78 |
|  | Comprehensive features | **0.88** | **0.88** | **0.94** | **0.85** | **0.92** | **0.91** | **0.86** |
| Test | Laboratory indicators | 0.78 | 0.77 | 0.87 | 0.62 | 1.00 | 1.00 | 0.67 |
|  | TCM pattern characteristics | 0.84 | 0.83 | **0.97** | 0.71 | 1.00 | 1.00 | 0.73 |
|  | Comprehensive features | **0.95** | **0.95** | 0.91 | **0.90** | 1.00 | 1.00 | **0.89** |

## Supplementary Figures


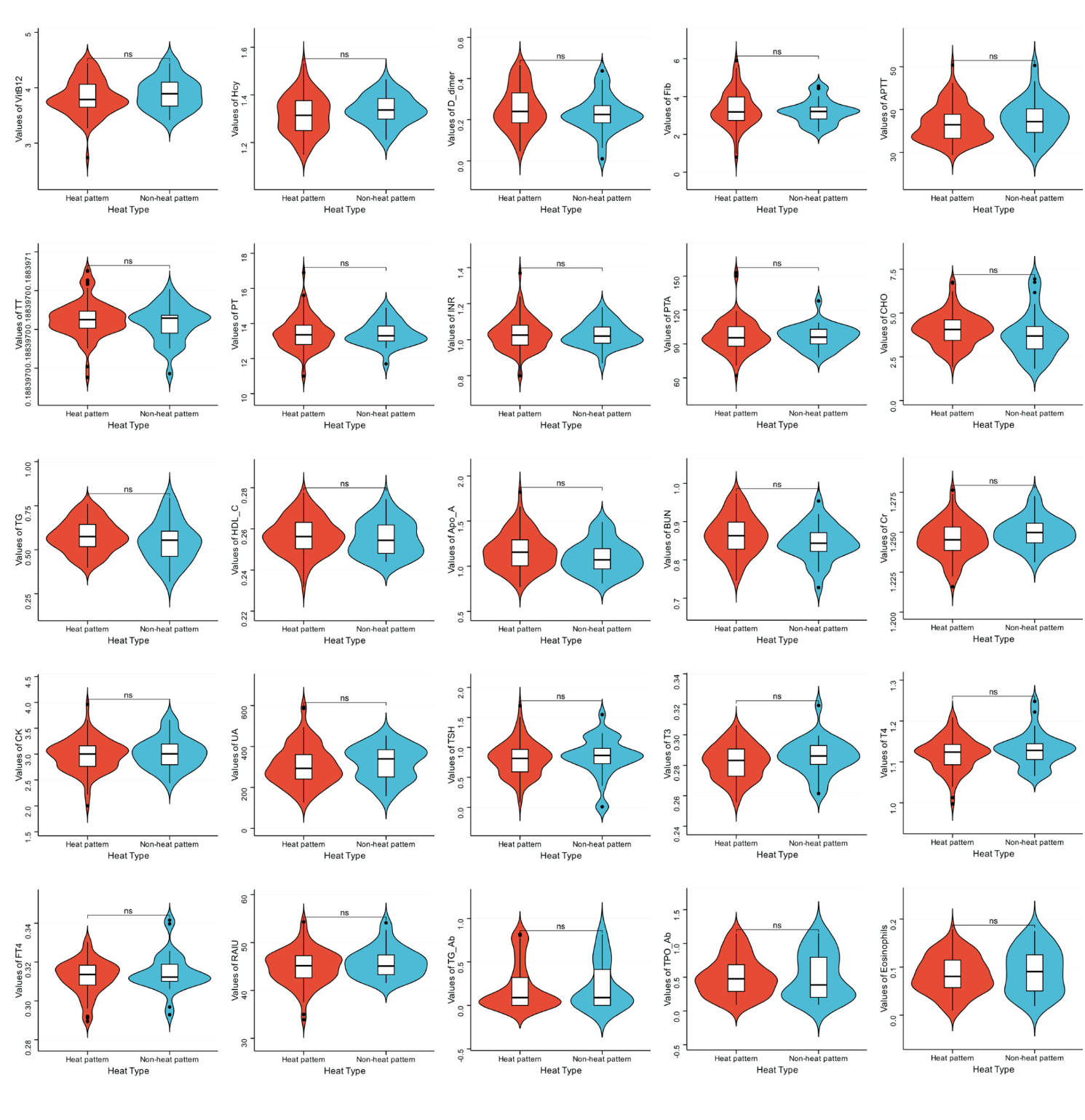
**Supplementary Figure 1.** Distribution of laboratory indicators in patients with heat pattern and non-heat pattern. Only indicators without statistically significant differences between the two are shown.


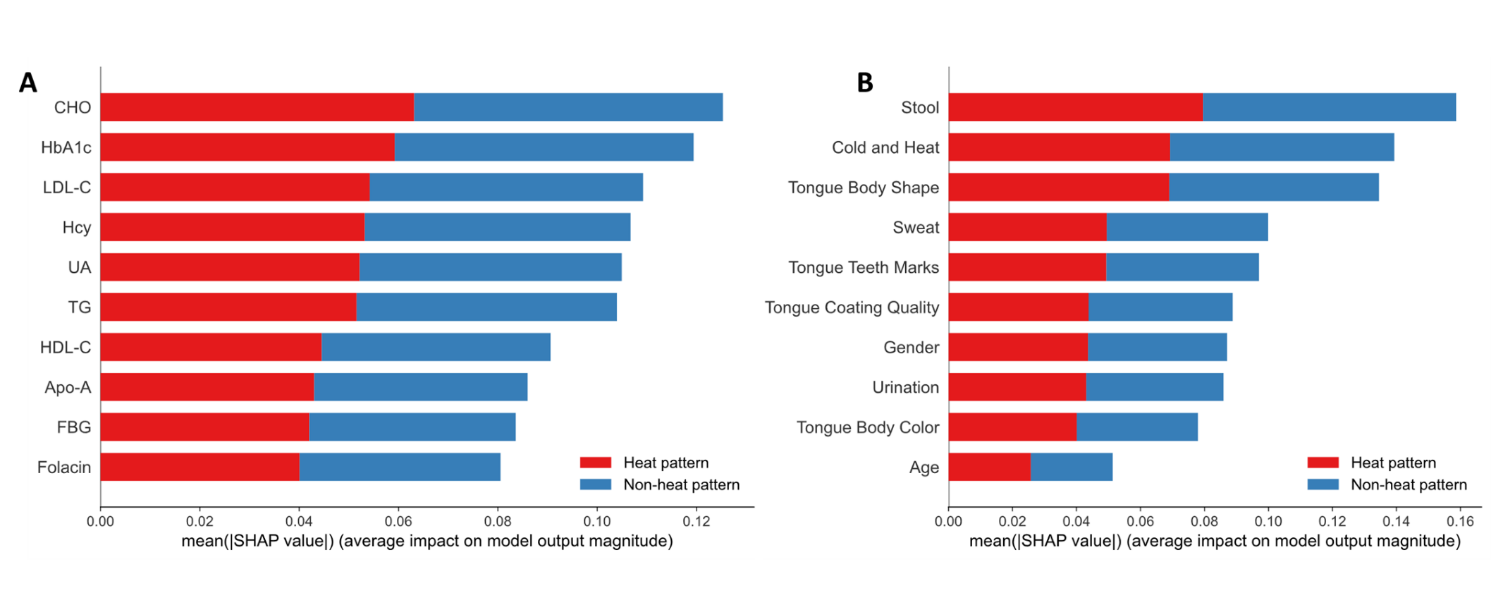


**Supplementary Figure 2.** Feature importance of the top 10 features using the average of the absolute SHAP values of two classes and ranking features in order of importance. (A) The importance of features with laboratory indicators data on the prediction results. (B) The importance of features with TCM pattern characteristics data on the prediction results.
